# The Variation of Duck RIG-I-Mediated Innate Immune Response Induced by Different Virulence Avian Influenza Viruses

**DOI:** 10.3389/fmicb.2022.842721

**Published:** 2022-03-01

**Authors:** Boyu Zhai, Lanlan Liu, Xiang Li, Xinru Lv, Jinyan Wu, Jing Li, Shengze Lin, Yuxiang Yin, Jiaqi Lan, Jianan Du, Chenwei Wu, Yi Wen, Yajun Wang, Yulong Wang, Zhijun Hou, Yanbing Li, Hongliang Chai, Xiangwei Zeng

**Affiliations:** ^1^State Forestry Administration Key Laboratory of Wildlife Conservation, College of Wildlife and Protected Area, Northeast Forestry University, Harbin, China; ^2^College of Basic Medical Science, Heilongjiang University of Chinese Medicine, Harbin, China; ^3^Chinese Academy of Agricultural Sciences Harbin Veterinary Research Institute, Harbin, China

**Keywords:** AIV, H5N8, H4N6, duck, RIG-I, IFN-β, lung, rectum

## Abstract

In recent years, the emerging highly pathogenic avian influenza (HPAI) A(H5N8) virus has been reported with features of widely spread, an expanding host range, and cross-species transmission, attracting wide attention. The domestic duck plays a major role in the epidemiological cycle of the HPAI H5N8 virus, but little is known concerning innate immune responses during influenza infection in duck species. In this study, we used two wild-bird-origin viruses, H5N8 and H4N6, to conduct duck infection experiments, and detect the load of the two viruses, and retinoic acid-inducible gene I (RIG-I) and interferon β (IFN-β) in the host’s natural immune response. Through comparison, it is found that the expression levels of RIG-I and IFN-β are both fluctuating. The innate immunity starts rapidly within 6 h after infection and is inhibited by the virus to varying degrees. The expression of RIG-I and IFN-β decreased on 1–2 days post-infection (dpi). The HPAI H5N8 virus has a stronger inhibitory effect on RIG-I than the low pathogenic avian influenza (LPAI) H4N6 virus and is the strongest in the lungs. After infection with HPAI H5N8 virus, 2 dpi, viral RNA replicates in large amounts in the lungs. It has been proven that RIG-I and IFN-β play an important role in the innate immune response of ducks to HPAI H5N8 virus infection, especially in the lungs. The main battlefield of RIG-I and IFN-β after infection with the LPAI H4N6 virus is in the rectum. Both viruses have been effectively controlled after 7 dpi. These results will help to understand the transmission mechanisms of avian influenza virus in wild ducks and help effectively prevent and control avian influenza.

## Introduction

Avian influenza, a highly contagious respiratory viral disease caused by the avian influenza virus (AIV), continues to impair the birds and human public health with enormous economic losses alarmingly worldwide. According to the antigenic difference between surface hemagglutinin (HA) and neuraminidase (NA), AIV is divided into 18 different HA subtypes and 11 different NA subtypes (Tong et al., 2013). According to the pathogenicity of AIV to chickens, it is divided into highly pathogenic avian influenza virus (HPAIV) and low pathogenic avian influenza virus (LPAIV).

Wild birds, especially waterfowl, are considered the natural hosts of AIV and the source of influenza viruses in other hosts (Webster et al., 1992). Except for H17N10 and H18N11, which are currently only isolated from bats (Zhu et al., 2013; Wu et al., 2014), almost all subtypes of AIV can be isolated from wild waterfowl (Piaggio et al., 2012; Zhang et al., 2012). Therefore, wild birds play an important role in the continued existence and spread of AIV. The role of wild birds in spreading LPAIV is recognized, and the situation of wild birds spreading HPAIV is more complicated. Studies have found that some wild birds (such as whooper swans, cormorants, bar-headed geese, and grebes; Spackman et al., 2009; Okamatsu et al., 2010; Albini et al., 2014; Muzyka et al., 2019) will die on a large scale and quickly after being infected with HPAIV, so they have little effect on the long-distance transmission of the virus. Wild ducks (such as mallards) are less likely to die after being infected with HPAIV and seem to have obvious resistance to HPAIV, which many studies have also confirmed (Sturm-Ramirez et al., 2005; Lee et al., 2016; Pantin-Jackwood et al., 2016). Therefore, wild ducks can migrate long distances in an asymptomatic state, and within a certain time frame, the virus can be excreted with their feces to cause influenza transmission.

Avian influenza viruses show obvious differences in virulence to different species of wild birds. Wild ducks show obvious tolerance. What is the molecular mechanism behind it? Immunity is an important barrier for keeping animals healthy, and especially, the natural immune response is the first line of defense against pathogenic infections. RIG-I is considered the most important pattern recognition receptor that initiates the body’s innate immune response during influenza virus infection. Due to the complex living environment of wild ducks, the infection background is unclear. The basic level of natural infection of different individuals varies. Therefore, this study selected domestic ducks artificially bred from mallard ducks as the model animal. The wild-bird-origin HPAIV caused the global H5N8 avian influenza epidemic in 2020–2021 (A/Whooper swan/Henan/h4/2016_H5N8), common wild bird LPAIV (H4N6) is used as the research object. Explore the differences in the innate immune changes caused by HPAIV and LPAIV in duck respiratory tract, intestines, and immune organs, through the infection experiment on SPF ducks, from the direction of RIG-1 mediated innate immunity. Try to analyze the possible molecular mechanism of duck innate immunity in influenza virus infection tolerance.

## Materials and Methods

### Virulent Strains

Two viruses were used for the animal study. An H5N8 virus, termed A/Whooper swan/Henan/h4/2016 (Hn/h4), was isolated from a dead whooper swan in November 2016 Henan Province, China. Another H4N6 virus, termed A/Shorebird/Liaoning/d59/2018 (Ln/d59), was isolated during our active surveillance of AIVs in April 2018, in Liaoning Province, China. Viruses were isolated with 9-10-day-old specific-pathogen-free (SPF) embryonated chicken eggs at National Avian Influenza Reference Laboratory of Harbin Veterinary Research Institute. Allantoic fluid was harvested after 72 h incubation, and the hemagglutinin (HA) activity was assayed. A hemagglutinin inhibition (HI) assay was performed preliminarily to determine the HA subtype of the isolated virus. Viral RNA was extracted from HA positive samples from the incubated allantoic fluid using a QIAamp Viral RNA Mini Kit (QIAGEN, Germany), reverse transcribed using the primer Uni12. The PCR products of eight fragments of the isolates were sequenced using Sanger Sequencing. The sequence data were compiled using the SeqMan program (DNASTAR, Madison, WI, United States). Hn/h4 possessed multiple basic amino acids (-RERRRKR↓GLF-) at the HA cleavage site, characteristic for HPAIV. Ln/d59 was classified as LPAI due to the -PEKASR↓GLF-amino acid motif.

### Animals

SPF Shaoxing Ducks were used as experimental animals. All ducks were hatched and raised until they reached within 720–800 g body weight at 4 weeks of age at the Negative pressure isolator in Laboratory Animal Center of Harbin Veterinary Research Institute.

### Animals Infection Experiment

The 50% egg infective doses (EID50) of the harvested allantoic fluids of Hn/h4 and Ln/d59 were determined by the method of Reed and Muench. Ninety SPF ducks were divided into three equal groups containing 30 ducks each. These groups were intranasally and orally inoculated with 0.2 ml 10^6^ EID50 of Hn/h4 (HPAIV group), Ln/d59 (LPAIV group), and Phosphate-buffered saline (PBS; control group), respectively. Three ducks of each group were randomly selected and humanely euthanized at the 1/12, 1/4, 1, 2, 3, 5, 7, and 10 dpi. Rectum, lung, and spleen were collected aseptically, dead ducks were selected priority, and all samples were frozen and stored at −80°C until total RNA extraction.

### Quantitative Real-Time PCR

Total RNA was extracted using Column Animal RNAout kit (Tiandz). The total RNA was reverse transcribed into cDNA with the primer oligo-dT for RIG-I and IFN-β expression determination by the relative quantitative method. The specific quantitative primers were designed using Oligo7 software, as followed, RIG-I (forward: qRIG-IF: 5′-GATACTCTCTCCCAAAACAGCAAGAAAGAT-3′ and reverse: qRIG-IR: 5′-GAAAAGGGCTCTACATATCCTGC -3′), IFN-β (qIFN-βF: 5’-CCCCGCAACCTTCACCT-3′ and qIFN-βR: 5′-CCGAAGTGGCTGGGAGATGC-3′), and β-actin (qβ-actinF: 5′-GCAAGTACTCTGTCTGGATTGGAG-3′ and qβ-actinR: 5′-TTTGCGGTGGACAATGGA-3′). Quantitative real-time PCR (qPCR) was performed on an ABI-7500 using TB Green™ Premix Ex Taq™ (Tli RNaseH Plus; Takara). The qPCR mixture consisted of 1 μl of cDNA sample, 3 μl nuclease-free water, 5 μl of 2 × TB Green Premix Ex Taq II(Takara), 0.4 μl of each gene-specific primer (10 mM), and 0.2 μl of ROX II(Takara). The qPCR cycling conditions were as:1 cycle of 95°C for 30 s, followed by 40 cycles of 95°C for 5 s, 60°C for 34 s.

The total RNA was reverse transcribed into cDNA with the primer Uni12 for viral loads determination by the absolute quantitative method. The specific quantitative primers were designed using Oligo7 software, as followed, H5N8 AIV (qH5N8F: 5′-GACTGGTTCATGCTCATGCC-3′ and qH5N8R: 5′-ACGGTGAGATTTCTCCCACAA-3′) and H4N6 AIV (qH4N6F: 5′-CTCTCGTTATTGCCGCAAGC-3′ and qH4N6R: 5′-TCAGGCACTCCTTCCGTAGA-3′). To prepare standard curves, we cloned small fragments of the two viruses into pMD^®^ 18-T vector (Takara), respectively, and verified by Sanger sequencing. PCR efficiency (E) was tested *via* the use of standard curves, using the formula E% = 10^(−1/slope)^ × 100, with 90–110% efficiency considered adequate for the purposes of this study. The qPCR procedure is the same as the relative quantification method described above.

### Statistical Analysis

The threshold cycle (Ct) value was normalized to the house-keeping gene β-actin; the expression levels of each gene were normalized to the expression level of the endogenous control (β-actin) and were expressed as fold changes relative to the control group using the 2^-ΔΔCt^ method (Livak and Schmittgen, 2001). Relative gene expression (i.e., fold change) was then calculated as the geometric mean of expression in each group (i.e., time point), with the experimental time points (1/12, 1/4, 1, 2, 3, 5, 7, and 10 dpi) scaled in comparison with the control group (1/12 dpi). Expression in the control group was set to 1 and relative fold change in target genes was expressed as positive values for upregulated genes and negative values for downregulated genes. Statistical comparison of changes in gene expression use t-text analysis. Viral loads were detected by qPCR with absolute quantification. Their respective copy numbers were calculated based upon the molecular weight of the plasmid for standards. Copy numbers for each viruses were then calculated based upon Ct values compared to standard curve. Statistical analyses and data plotting were performed with GraphPad Prism v. 8.0.1 (GraphPad Software Inc.).

## Results

During the animal experiment, two ducks died in the HPAIV group, one on the 2nd day and the other on the 5th day, and there was no death in the LPAIV group and the control group. There were no obvious clinical symptoms in all groups. Through planning inspection, it was found that ducks in the HPAIV group had light yellow mucus in the trachea, flushing of the lungs, and swelling of the spleen to varying degrees as the number of days increased. Only the spleen was swollen in the LPAIV group. The control group was healthy and free of symptoms.

**Figure 1:**
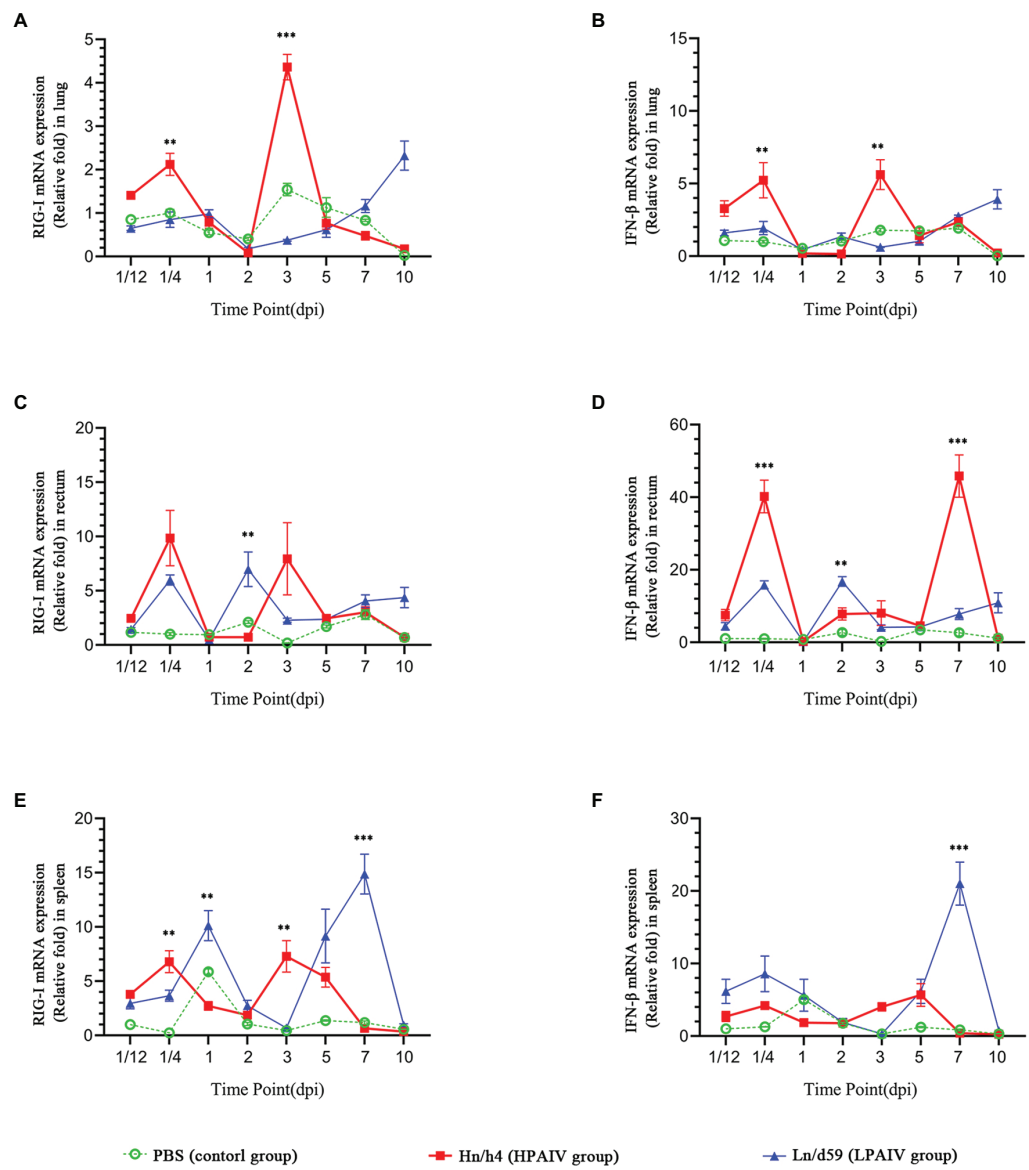
Changes in the expression levels of RIG-I and IFN-β in different tissues. **(A)** Relative expression of RIG-I in the lung. **(B)** Relative expression of IFN-β in the lung. **(C)** Relative expression of RIG-I in the rectum. **(D)** Relative expression of IFN-β in the rectum. **(E)** Relative expression of RIG-I in the spleen. **(F)** Relative expression of IFN-β in the spleen. Asterisks represent significant changes (*t*-test), ^**^represents *p* < 0.01, and ^***^represents *p* < 0.001.

**Figure 2:**
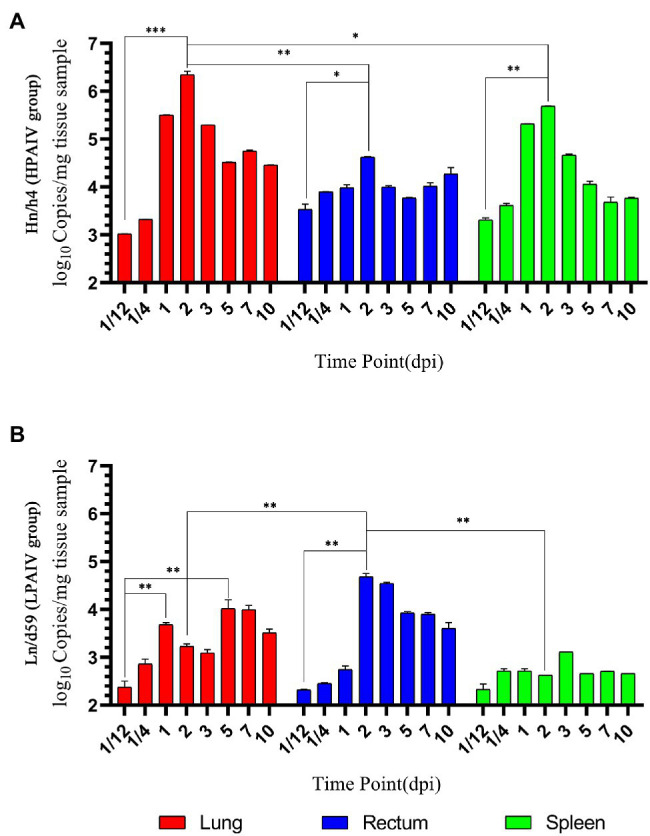
Changes in viral load. **(A)** Copy number of Hn/h4 at different time points. **(B)** Copy number of Ln/d59 at different time points. Asterisks represent significant changes (*t*-test), ^*^represents *p* < 0.5, ^**^represents *p* < 0.01, and ^***^represents *p* < 0.001.

According to the analysis of qPCR results, the relative expression change curves of RIG-I and IFN-β and the copy number of the virus were drawn. In duck lungs (Figures 1A,B), Hn/h4 will cause a short rise of RIG-I before 1/4 dpi, and then, the expression of RIG-I will drop below normal within 2 dpi, then start to rise and reach a peak at 3 dpi, ultimately, return to normal level in 10 dpi. The expression of IFN-β is the same as the changing trend of RIG-I. Compared with the LPAIV group, the changing trend of RIG-I and IFN-β expressions was delayed, and the peak appeared later. Ln/d59 only slightly increased at 1 and 5 dpi after infection (Figure 2B). After the challenge, Hn/h4 started to replicate in large quantities 1 dpi (Figure 2A). The content of viral RNA in the lungs was at a high level within 2 dpi, which was extremely significantly different from the 1/12 dpi. (*p* < 0.001). After 3 days, Hn/h4 was inhibited and began to decline rapidly but Ln/d59 was rise again in 5 dpi.

After challenge with Hn/h4, RIG-I expression in the rectum of ducks was similar to that in the lungs (Figures 1C,D). Unlike the lungs, the highest peak of RIG-I expression in the rectum appeared at 1/4 dpi instead of 3 dpi. The expression of IFN-β was highly upregulated before 1/4 dpi, decreased below the normal level at 1/4–1 dpi, and began to rise after 1 dpi. The changing trend of RIG-I and IFN-β is high expression at 1/4 dpi, and the expression level rises briefly and rapidly. The changing trend of RIG-I and IFN-β expressions caused by Ln/d59 in the rectum is the same, and it is also in the rising stage before 1/4 dpi, but both are lower than Hn/h4. Two peaks are formed at 1/4 dpi and 2 dpi. The highest copy number of Hn/h4 in the rectum appears at 2 dpi, which is not significantly different from 1/12 dpi (*p* < 0. 5; Figure 2A). The copy number of Ln/d59 increased rapidly at 2 dpi and then decreased slowly and the peak copy number was significantly different from 1/12 dpi (*p* < 0.01; Figure 2B).

The changing trend of RIG-I expression in the spleen of ducks infected with Hn/h4 is consistent with that of the lung and rectum (Figures 1E,F). The highest peak of IFN-β expression appeared at 5 dpi after the challenge. Compared with Ln/d59, the peak expression of RIG-I and IFN-β appeared earlier and lower. The highest peak of RIG-I and IFN-β expression after infection with Ln/d59 appeared at 7 dpi. The copy number of Hn/h4 increases rapidly after 1/4 dpi and peaks at 2 dpi and is significantly different from 1/12 dpi (*p* < 0.01; Figure 2A). The copy number of Ln/d59 in the spleen was low, and there was no obvious change, only a slight increase at 3 dpi (Figure 2B).

**Figure 3:**
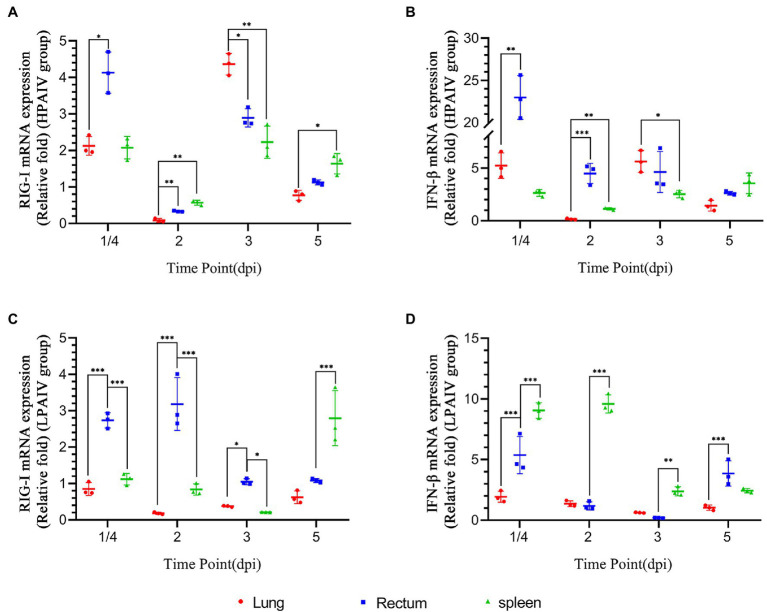
Comparison of the expression levels of RIG-I and IFN-β in different tissues at specific time points. **(A)** The relative expression of RIG-I in different organs simultaneously after Hn/h4 infection. **(B)** The relative expression of IFN-β in different organs simultaneously after Hn/h4 infection. **(C)** The relative expression of RIG-I in different organs simultaneously after Ln/d59 infection. **(D)** The relative expression of IFN-β in different organs simultaneously after Ln/d59 infection. Asterisks represent significant changes (*t*-test), ^*^represents *p* < 0.5, ^**^represents *p* < 0.01, and ^***^represents *p* < 0.001.

The representative time points were selected, and the expression levels of various genes in duck lung, rectum, and spleen simultaneously were compared based on the data. The expression of RIG-I in duck lungs infected with Hn/h4 was higher than that of rectum and spleen at 3 dpi, and low expression at 1/4, 2, and 5 dpi. In the intestine, the expression of RIG-I was highest at 1/4 dpi (Figure 3A). The situation of IFN-β is similar to that of RIG-I, only slightly different at 2 dpi (Figure 3B). The expression of RIG-I in ducks infected with Ln/d59 was highest in the rectum at 1/4, 2, 3 dpi, especially at 1/4, 2 dpi, and the lung and spleen were significantly different, while at 5 dpi, it was the highest in the spleen (Figure 3C). The expression level of IFN-β is opposite to that of RIG-I. The expression level of IFN-β is the highest in the spleen at 1/4, 2, and 3 dpi and the highest in the intestine at 5 dpi (Figure 3D). After infection with Ln/d59, both RIG-I and IFN-β are low expression in the lungs.

## Discussion

### The Spread of HPAIV and LPAIV Among Wild Birds

Studies have shown two main ways of transmission of AIV in wild birds. The virus infects the upper respiratory tract and then spreads to other hosts through droplets. The other is that the carrier excretes the virus into the water through excretion, and AIV uses water as a medium to spread to other hosts. AIV can maintain infectivity for more than 1 month in cold water and 4 days at room temperature (Webster et al., 1978).

The main replication site of HPAIV is in the upper respiratory tract of the host, and it is highly lethal to wild birds (Sturm-Ramirez et al., 2004). Most migratory birds cannot carry HPAIV for long-distance migration. The replication site of LPAIV is mainly in the intestine (Webster et al., 1978), which can be transmitted through the water as a medium, and most wild birds are not lethal after infection, and even no obvious symptoms will appear (Kida et al., 1980). For these reasons, LPAIV spreads across regions through the migration of wild birds. LPAIV is easy to mutate and recombine with other AIVs in the host body (Holmes et al., 2005; Rambaut et al., 2008; Marshall et al., 2013), which further enhances its virulence and transforms it into a more harmful HPAIV (Zhang et al., 2013).

At present, the AIV of the H5N8 subtype has gradually replaced the dominant position of H5N1 and has gradually become the dominant virus in the world, and cause a large number of wild birds to infect and die especially Anseriformes. A large amount of epidemiological and genetic evidence shows that wild ducks are not dying after being infected with the HPAI H5N8 virus and can carry the virus to spread over long distances (Global Consortium for H5N8 and Related Influenza Viruses, 2016; Nuñez and Ross, 2019). This situation was also confirmed in this experiment. The ducks did not die in large numbers after being infected with Hn/h4, and there were no obvious symptoms. Therefore, wild ducks have important research value in transmitting the HPAI H5N8 virus. Because of the H4N6 subtype, AIV is the most common type of LPAIV, and it has a wider range of transmission. It is often isolated from wild waterfowl in many countries in Asia, Europe, Africa, and North America (Nguyen et al., 2005; Simulundu et al., 2011). Therefore, Hn/h4 (H5N8) and Ln/d59 (H4N6) were chosen to analyze the immune mechanisms between AIVs with different virulence, which will build the fundamental theoretical basis to prevent the cross-regional spread of HPAIV and LPAIV among wild birds.

### The Important Role of RIG-I in Resisting Avian Influenza Virus

Innate immunity is the body’s first barrier encountered by invading AIVs, and virus infection is detected by identifying pathogen-associated molecular patterns (PAMP) through pattern recognition receptors (PRR). These PAMPs are produced during pathogen infection (Janeway, 1989). Members of at least three different types of PRRs recognize influenza viruses through the innate immune system, namely, Toll-like receptors (TLRs), RIG-1-like receptors (RLRs), NOD-like receptors (NLRs), and C-type lectins Receptors (CLRs; Kawai and Akira, 2009). Among them, RLRs mainly include RIG-I, melanoma differentiation-associated gene 5 (MAD5), and Laboratory of genetics and physiology 2 (LGP2; Takeuchi and Akira, 2010).

Currently, RIG-I is recognized as one of the main pattern recognition receptors (PRR) for innate immunity against influenza viruses. RIG-I is activated by binding to viral RNA, causing conformational changes to activate downstream pathways. IKKα and IKKβ lead to the activation of nuclear factor-κB (NF-κB; Servant et al., 2002; Hayden and Ghosh, 2011), and phosphorylation mediated by IKKɛ and TBK1 activates interferon regulatory factor 3/7 (IRF3/7; Sharma et al., 2003), which can cause the production of interferon, interferon-stimulating genes, and pro-inflammatory cytokines, and block the replication of the virus in the body (Goodbourn et al., 2000).

According to research, RIG-I does not exist in chickens (Barber et al., 2010). Although chickens without RIG-I can also produce interferon-α (IFN-α) through other means, the expression of IFN-β during influenza virus infection is largely dependent on RIG-I (Isaacs and Lindenmann, 1987). From the experimental data, the expression transformation trend of RIG-I and IFN-β does have high similarity. The protective effect of IFN-β during influenza infection cannot be replaced by IFN-α (Koerner et al., 2007; Barber et al., 2010). This results in that chickens are less resistant to AIV than ducks, and RIG-I plays a decisive role in it.

### Links Between Viral Load and Innate Immunity

Studies have shown that AIV preferentially replicates in different tissues of the host, initially often depending on the type of linkage of cell surface glycoproteins. It is also specific in different tissues (Campbell and Magor, 2020). Usually HPAIV is more adaptable in the upper respiratory tract, and LPAIV is more adaptable in the intestine (Webster et al., 1978; Kida et al., 1980; Sturm-Ramirez et al., 2004; Vanderven et al., 2012). However, viral load cannot be explained by target cell limitation alone; there are also other factors such as innate immunity (Hagenaars et al., 2016). The distribution and expression changes of innate immune genes in tissues also have influence on them. There are also differences in innate immunity elicited in tissues that replicate heavily after viral infection. This may be due to mutations in the amino acid sites of the virus, resulting in different virulence (Suttie et al., 2019). Generally, the innate immune response caused by HPAIV is more intense and that caused by LPAIV is milder (Fleming-Canepa et al., 2019). The link between viral load and innate immunity may be more complex than we currently understand. Further research is therefore required.

### Similar Innate Immune Response Caused by AIV of Different Virulence

The data show that both viruses began to replicate in large numbers at 1–3 dpi and were effectively controlled after 7 dpi, which may be because the innate immune gene expression level is not high at 1–2 dpi, and the expression levels of RIG-I and IFN-β are in a decline stage. After 7 dpi, adaptive immunity begins to take effect, and it usually takes about a week to produce antibodies (Kikkert, 2020). A large number of antibodies inhibited the replication of AIVs. At the same time, this also illustrates the important role of innate immunity against the avian influenza virus in the 7 days before infection.

The results showed a strong correlation between the expression changes of RIG-I and IFN-β caused by the two AIVs with different pathogenicity. The expression of RIG-I and IFN-β increased temporarily at 6 h after infection. According to research, 12 h after H5N6 avian influenza virus infection, the expression of RIG-I in ducks is also upregulated (Wu et al., 2019). The expression of RIG-I in the lungs of ducks infected with H9N2 AIV was also slightly upregulated (Cheng et al., 2015). These research results are consistent with the results of this experiment, but the time nodes of the experimental design are not intensive enough to accurately reflect how long the upward trend will continue. Innate immunity is the body’s first barrier. Once a virus invades it, an immune response will occur quickly, preventing virus replication in a short period.

Interestingly, the expression of RIG-I and IFN-β after infection with AVIs did not increase or decrease continuously but fluctuated. We speculate that this may be because downstream signals negatively regulate RIG-I to ensure that the innate immune response is strong enough but not too explosive. When the innate immunity is too strong, these responses are downregulated in time to protect the individual from damage (Robertson, 1998). It may also be due to the suppressive effect of the virus on innate immunity.

### Differences in Innate Immune Response Caused by AIVs of Different Virulence

After a brief rise, the innate immunity genes may be suppressed. During the 1–2 dpi after challenge with Hn/h4, RIG-I and IFN-β expressions will continue to decline until they are lower than normal levels. It may be due to the interference of the virus’s NS1 protein on the RIG-I pathway. NS1 protein can form a complex with RIG-I, causing RIG-I to be unable to be activated (Pichlmair et al., 2006; Guo et al., 2007; Mibayashi et al., 2007; Opitz et al., 2007). At the same time, it can also hinder the formation of IRF3/7 (Talon et al., 2000) and NF-κB (Wang et al., 2000), resulting in blocked signal pathways and unable to stimulate the formation of IFN. The NS1 protein can also inhibit the post-transcriptional processing and modification of host cell precursor mRNA from affecting IFN production (Kuo and Krug, 2009) so that the virus has a period of concealed replication after infection (Vanderven et al., 2012). The virus replicated in large numbers at this point and reached a peak in 2–3 dpi. The relationship between the virulence of the virus and the degree of influence of the NS1 protein on innate immunity is not clear. However, from the data results, Hn/h4 has a stronger inhibitory ability on the innate immune gene RIG-I pathway than Ln/d59.

Interestingly, the experimental data showed that the inhibitory ability of Hn/h4 in the lung is stronger than that in the spleen and rectum. It may be because the main replication site of HPAIV is in the respiratory tract (Sturm-Ramirez et al., 2004; Vanderven et al., 2012), so it may show stronger inhibitory ability in the lung. Studies have shown that compared with the low pathogenic H5N1 AIV, the highly pathogenic H5N1 AIV inhibits the immune response in the respiratory tract by inhibiting the C-X-C chemokine receptor 4 (CXCR4) signaling pathway and promotes systemic infection in ducks (Massin et al., 2013). Some researchers have shown that after infection with low pathogenic H5N1 AIV, RIG-I expression increased significantly in the lungs on the first day (Barber et al., 2010). However, the expression of RIG-I in the lung after infection with the Hn/h4 HPAIV in this experiment showed a downward trend, and the expression was lower than that of the spleen and rectum at 2 days (Figure 3). This result shows that Hn/h4 may also inhibit the CXCR4 signaling pathway and shows a stronger inhibitory effect on RIG-I in the lungs. In addition, the copy number of Hn/h4 in the rectum at 1/4 dpi is higher than that in the lung and spleen, and the upregulation of RIG-I and IFN-β expression is caused by it is also the highest. The replication of Hn/h4 is effectively controlled in the rectum, and there is no sudden sharp rise, showing that Hn/h4 does not exert a strong inhibitory effect on innate immunity in the rectum as it does in the lung.

The results showed that as the copy number of Ln/d59 increased in the rectum, the expression of RIG-I and IFN-β also increased. They effectively resisted Ln/d59 and successfully prevented the virus from replicating. The copy number of Ln/d59 in the lung and spleen is lower than that in rectum, especially in the spleen, which is basically at the detection limit. Although Ln/d59 caused little change in the expression of RIG-I and IFN-β in the lung, it was different in the rectum and spleen. The expression of RIG-I in the intestine is significantly different from that in the lung and spleen at 1/4, 2, and 3 dpi. The expression of IFN-β in the spleen was higher than that in the rectum and the lung at these time points, and there were significant differences (Figure 3D), which may be because the main replication site of Ln/d59 is in the rectum, which has the same characteristics as other LPAIVs (Webster et al., 1978; Kida et al., 1980), and RIG-I in ducks is mainly distributed in the mucosal tissues in contact with air (Cheng et al., 2015). The spleen is an immune organ, and multiple immune pathways may cause the high expression of IFN-β (John, 1994). Studies have shown that the spleen plays a particularly active role in the innate immune response to LPAI, which is consistent with the data from this experiment (Helin et al., 2018).

In summary, RIG-I and IFN-β play an important role in the fight against H5N8 and H4N6 AIVs in ducks. Especially within a few hours after infection, the upregulation is rapid. Although the virus inhibits it within 1–2 days, it is eventually upregulated and hinders the increase in viral load. These two AIVs have different main replication sites, but H5N8 has a stronger ability to inhibit innate immunity than H4N6. Recently, there have been reports that many wild whooper swans and black-necked gray swans died after being infected with the H5N8 avian influenza virus (Muzyka et al., 2019; Li et al., 2021, 2022). We want to analyze further the causes of their deaths and the changes in RIG-I after infection with the H5N8 avian influenza virus. In the experiment, it can be seen that AIVs of different virulence will have significantly different effects on innate immunity. Will there be obvious differences in the resistance of different breeds of ducks to AIVs? We still need further research and discussion about whether there are other common rules in the interaction between AIVs and the body’s immunity. In recent years, the pathogenicity of AIVs has been continuously enhanced, and the host range has become wider and wider, and at the same time, it continues to pose a threat to mammals, especially humans. However, the biggest problem is how to prevent AIVs from spreading across regions in wild birds. This study compared the differences in the innate immune mechanism after HPAIV and LPAIV infection and analyzed their transmission mechanisms. It is hoped that it can provide a theoretical basis for the large-scale spread of AIVs and help prevent, control, and treat AIVs in the future.

## Data Availability Statement

The original contributions presented in the study are included in the article/supplementary material, further inquiries can be directed to the corresponding authors.

## Ethics Statement

The animal study was reviewed and approved by Harbin Veterinary Research Institute, Chinese Academy of Agricultural Sciences, Heilongjiang, China.

## Author Contributions

BZ, LL, and XLi performed the phylogenetic analyses and the animal experiments and wrote the drafts of the manuscript. Xlv, JW, JLi, SL, YY, and JLa collected the surveillance samples and performed the Quantitative real-time PCR. JD, CW, and YWe contributed to isolation and identification of virus. YWa, YLW, and ZH performed the phylogenetic analyses. YL, HC, and XZ commented on and revised the drafts of the manuscript. All authors contributed to the article and approved the submitted version.

## Funding

This study was supported by the Heilongjiang Natural Science Foundation Program (LH2019C13) and the Surveillance of Wildlife Diseases from the National Forestry and Grassland Administration (2019076018) and the National Natural Science Foundation of China (81873312).

## Conflict of Interest

The authors declare that the research was conducted in the absence of any commercial or financial relationships that could be construed as a potential conflict of interest.

## Publisher’s Note

All claims expressed in this article are solely those of the authors and do not necessarily represent those of their affiliated organizations, or those of the publisher, the editors and the reviewers. Any product that may be evaluated in this article, or claim that may be made by its manufacturer, is not guaranteed or endorsed by the publisher.
